# Thumb hypoplasia: current concepts

**DOI:** 10.1186/1753-6561-9-S3-A11

**Published:** 2015-05-19

**Authors:** Michael Tonkin

**Affiliations:** 1 Department of Hand Surgery, University of Sydney, New South Wales, 2006, Australia

## 

This presentation discusses an updated classification of thumb hypoplasia which allows greater relevance of comparison of surgical results; advises upon the indications for specific surgical techniques; presents some detail of many but not all surgical techniques; and discusses methods of assessment of outcomes following surgery.

The Blauth classification of hypoplastic grades 1-5 is based on the work of Mueller who proposed the concept of an increasing severity of hypoplasia of all thumb components, soft tissue and bone, with increasing severity of insult. Manske and others have sub-divided grade 3 in an attempt to compare like with like but this classification does alter the integrity of the Blauth system which distinguishes between grades 2 and 3 by the presence or absence of the proximal metacarpal. My proposed classification maintains the integrity of Blauth’s classification, sub-dividing within grade 2. Grade 2A is of mild nature with first web deficiency, uniaxial MCP joint instability and hypoplasia of intrinsic muscles, each of which may require reconstruction through local flaps in the first web, ulnar collateral ligament reconstruction and an opposition transfer. Extrinsic anomalies may be present but do not deserve attention. Grade 2B is moderate in nature and in this instance extrinsic anomalies which require reconstruction are present as is global MCP joint instability. An FDS transfer is the most appropriate opposition transfer as the tails of the tendon may be used for reconstruction of ulnar and radial collateral ligaments. A palmar plate advancement may assist in hyperextension of the MCP joint. A more extensive first web plasty with skin grafting may be necessary. Grade 2C is of severe nature with severe global MCP joint instability as demonstrated by the “elephant’s trunk” sign. Fusion of the MCP joint is appropriate but this does have the problem of increasing the length of the lever arm, placing greater stress across an unstable CMC joint. Furthermore, it shortens the digit, places the growth plate at risk and it may be possible to obtain a chondrodesis only if the epiphysis has not ossified. Regardless, it is almost impossible to reconstruct such joints with soft tissue procedures alone. The CMC joint is also inadequate, being unstable or immobile. Most current classifications do not attend to this complex problem. A radiological “pencil sign” is indicative of the severe underdevelopment of the CMC joint, which may respond to a soft tissue reconstruction. However, occasionally pollicisation may give superior outcomes for the severe grade 2C hypoplasias.

Distinction between grades 3A and 3B on the basis of the amount of the metacarpal absent, the proximal third in 3A and the proximal two-thirds in 3B, allows a comparison of surgical results when one decides to reconstruct these severely hypoplastic thumbs. Results of reconstruction tend to be improved when there is more to work with. For the same reasons the grade 4 thumb is less likely to be as functional as a grade 3A or 3B thumb when reconstruction of the floating digit is attempted.

Pollicisation remains the preferred option for grades 3, 4 and 5, and occasionally for grade 2C. A whole toe transfer is an option for reconstructing a grade 5 thumb when a determination to obtain five digits is absolute.

Assessment of results following reconstruction of the paediatric hypoplastic thumb is difficult. An assessment scale is offered which incorporates components of scales previously described by Percival and Japanese authors, not only following reconstruction of the hypoplastic thumb but also following reconstructions of thumb duplications. Although such an assessment scale contains some flaws, it does provide an objective basis from which pre- and post-operative comparisons can be made and comparisons between surgical results of one may be compared with those of others. To achieve this, it is necessary to begin with a classification which at least allows comparison of like with like.

